# Use of Radiomics to Improve Diagnostic Performance of PI-RADS v2.1 in Prostate Cancer

**DOI:** 10.3389/fonc.2020.631831

**Published:** 2021-02-17

**Authors:** Mou Li, Ling Yang, Yufeng Yue, Jingxu Xu, Chencui Huang, Bin Song

**Affiliations:** ^1^Department of Radiology, West China Hospital of Sichuan University, Chengdu, China; ^2^Department of Research Collaboration, R&D Center, Beijing Deepwise & League of PHD Technology Co., Ltd, Beijing, China

**Keywords:** artificial intelligence, radiomics, prostate cancer, multi-parametric MRI, PI-RADS v2.1

## Abstract

**Objective:**

To investigate whether a radiomics model can help to improve the performance of PI-RADS v2.1 in prostate cancer (PCa).

**Methods:**

This was a retrospective analysis of 203 patients with pathologically confirmed PCa or non-PCa between March 2015 and December 2016. Patients were divided into a training set (n = 141) and a validation set (n = 62). The radiomics model (Rad-score) was developed based on multi-parametric MRI including T2 weighted imaging (T2WI), diffusion weighted imaging (DWI), apparent diffusion coefficient (ADC) imaging, and dynamic contrast enhanced (DCE) imaging. The combined model involving Rad-score and PI-RADS was compared with PI-RADS for the diagnosis of PCa by using the receiver operating characteristic curve (ROC) analysis.

**Results:**

A total of 112 (55.2%) patients had PCa, and 91 (44.8%) patients had benign lesions. For PCa versus non-PCa, the Rad-score had a significantly higher area under the ROC curve (AUC) [0.979 (95% CI, 0.940–0.996)] than PI-RADS [0.905 (0.844–0.948), *P* = 0.002] in the training set. However, the AUC between them was insignificant in the validation set [0.861 (0.749–0.936) *vs.* 0.845 (0.731–0.924), *P* = 0.825]. When Rad-score was added to PI-RADS, the performance of the PI-RADS was significantly improved for the PCa diagnosis (AUC = 0.989, *P* < 0.001 for the training set and AUC = 0.931, *P* = 0.038 for the validation set).

**Conclusions:**

The radiomics based on multi-parametric MRI can help to improve the diagnostic performance of PI-RADS v2.1 in PCa.

## Introduction

Prostate cancer (PCa) remains the most commonly diagnosed malignancy among men in the western world (1). The frequency of PCa in Asia has increased rapidly in years (2). Accurate detection and diagnosis of PCa are key factors to improve its therapeutic response and prognosis. Currently, magnetic resonance imaging (MRI) is generally considered the best modality for the detection and localization of PCa, and are thus becoming increasingly important (3). A recently developed multi-parametric (mp) MRI protocols including T2-weighted (T2W), diffusion-weighted (DWI), and dynamic contrast-enhanced (DCE) imaging, appears to have good performance for PCa diagnosis, when associated with the Prostate Imaging Reporting and Data System (PI-RADS) (4).

In 2012, the initial version (v1) of PI-RADS was released to promote standardized MRI techniques and image interpretation. However, limitations of version 1 were soon evident. In 2015, version 2 was described to further improve reporting accuracy, and now has seen a broad uptake (4, 5). In 2019, PI-RADS version 2.1 was newly described, with several studies suggesting that version 2.1 could be preferable than version 2 for the evaluation of transition zone PCa (6–8). PI-RADS is now playing an increasingly prominent role in PCa diagnosis (9, 10). However, PI-RADS seems to have limitations of relatively low specificities, and inter-reader reproducibility. Thus a quantitative diagnostic method is needed to improve the performance of PI-RADS for the definite diagnosis of PCa (11, 12).

Radiomics can provide large-scale radiological image analysis by using a large number of quantitative features (13). Compared with genomics and proteomics, radiomics has the advantages of non-invasion assessments, comprehensive views of whole tumor and convenience in routine practice; thus, this technique has great potential for application in individualized diagnosis and treatment. Two studies (4, 14) have shown that radiomics can be used to detect PCa. However, it’s uncertain whether radiomics can add value to PI-RADS in the diagnosis of PCa. Therefore, this study aimed to determine whether radiomics of mpMRI can enhance the performance of PI-RADS v2.1 in PCa diagnosis.

## Materials and Methods

### Patients

This study was approved by the local Institutional Review Boards (No. 2019-1209, Date: December 30, 2019) and the need for written informed consent was waived.

**Figure 1:**
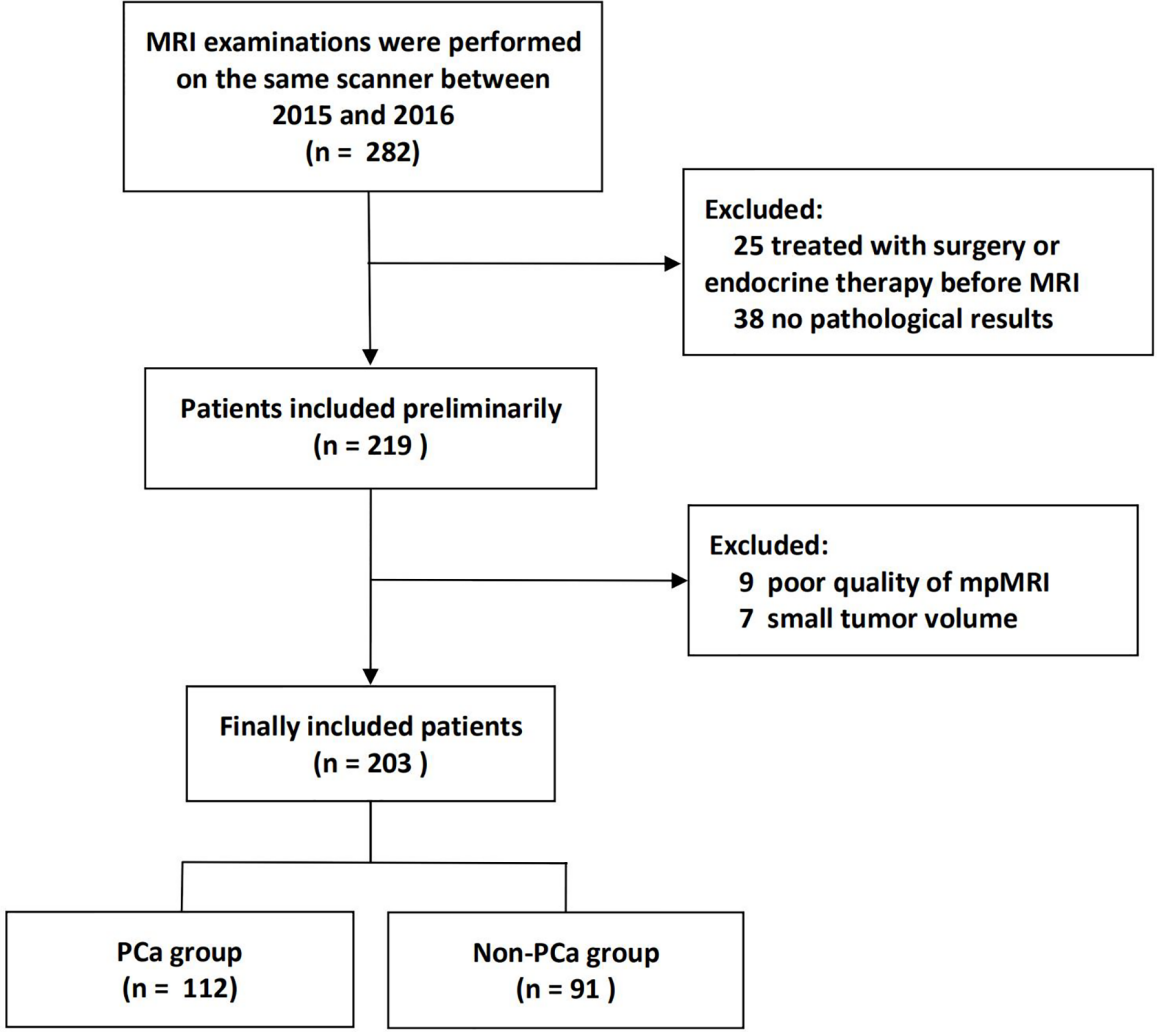
Flow chart of patients’ recruitment pathway.

The institutional database of medical records was searched for suitable patients between March 2015 and December 2016. A total of 203 patients (mean age 66 years, age range 36–85 years) who met the following criteria were finally enrolled. The inclusion criteria: (1) Men with suspicious lesions on mpMRI; (2) These lesions were histologically confirmed by biopsy or radical prostatectomy; (3) no prior prostate endocrine therapy, biopsy, surgery, or radiation therapy before MRI examination. The exclusion criteria: (1) lesions with maximum transverse diameter <5 mm, which could hardly be delineated on MRI; (2) poor mpMRI quality. The patient recruitment pathway was shown in **Figure 1**.

The baseline characteristics, pathological data, and radiographic evaluation of each patient, including age, size, location, Gleason score, PI-RADS v2.1 score and prostate-specific antigen (PSA) were shown in **Table 1**. The patients were divided into two groups (the training and validation sets) at a ratio of 7:3 according to the scanning date.

**Table 1:** Patient and tumor characteristics.

Characteristics	PCa (n = 112)	Non-PCa (n = 91)	*P* value	Training cohort (n = 141)	Validation cohort (n = 62)	*P* value
Age (y)	66.0 ± 7.6	66.0 ± 10.3	0.970	66.5 ± 8.6	64.9 ± 9.42	0.245
Size (long axis, mm)	2.22 ± 1.08	1.56 ± 0.88	0.001	1.86 ± 0.96	2.07 ± 1.21	0.194
Location			0.023			0.479
Transitional zone	51	56		72	35	
Peripheral zone	61	35		69	27	
PSA (ng/ml)			0.000			0.749
PSA ≤ 10	15	41		37	19	
10<PSA ≤ 20	31	37		47	21	
PSA>20	66	13		57	22	
Gleason score (6/7/8/9)	11/69/8/24	–		8/50/5/15	3/19/3/9	0.797
Extracapsular invasion on MRI (+/−)	74/38	6/85	0.000	58/83	22/40	0.448
PI-RADS v2.1 score			0.000			0.651
1	0	3		1	2	
2	0	26		18	8	
3	6	30		24	12	
4	32	25		42	15	
5	74	7		56	25	

This table summarized the integrated PI-RADS scoring results for the two readers. The weighted Kappa between two readers was 0.722 (95% CI 0.651–0.794), which indicated strong consistency.

PCa, prostate cancer; PSA, prostate-specific antigen.

### MRI Examination

All MRI examinations were performed on the same 3.0 T MRI scanner (Skyra, Siemens Healthcare Sector, Germany) with a pelvic phased array coil. Scan sequences included T2WI in the axial and sagittal planes, DWI with b values of 0, 200, 400, and 1,000 s/mm^2^, and DCE. ADC maps were calculated on a designated workstation. **Supplementary Material** summarizes the parameters of mpMRI sequences, including the type, repetition time/echo time (TR/TE), section thickness, field of view (FoV), and bandwidth.

### Reference Standard for Pathology

All lesions were histopathologically proven based on biopsy (transrectal ultrasound [TRUS]-guided 12-core systematic biopsy) or surgical specimens (radical prostatectomy). Pathological confirmatory reports were acquired from medical records of the Department of Pathology.

### PI-RADS Evaluation

Two experienced radiologists (more than 5 years of experience in the diagnosis of PCa) were assigned to review the mpMRI. The patient identification was removed from all images, and the readers were blinded to all clinicopathological information. The mpMRI including T2WI, DWI with corresponding ADC map, and DCE of the largest lesion in each patient was scored with a scale of 1–5 using PI-RADS v2.1. PI-RADS scores obtained by the two readers were assessed by a weighted Kappa statistics test to evaluate the inter-observer variability. Then any disagreement between the two readers was solved by discussion during the image interpretation.

### Texture Feature Extraction and Model-Building

The images were normalized before feature calculation. In detail, each image was subtracted by the mean value and was divided by the standard deviation value. Then the image was multiplied by 100, and resampled to the same resolution.

Two radiologists drew volume of interest (VOI) independently on MR images of 30 patients to evaluate the stability of the features. Only the features with inter- and intra-class correlation coefficient (ICC) > 0.75 can be included in the following analysis. The entire VOI of the tumor were drawn on the base of radiologic-histologic correlation slice by slice (the radiologists were blinded to the histopathology results). For the patient with multiple lesions, only the dominant lesion (the largest lesion) was segmented.

Radiomic features of the lesions were extracted using PyRadiomics. Three types of features (first-order statistics, texture features, and shape features) for a total of 1,304 features were extracted from each sequence of mpMRI. To eliminate the differences in the value scales of radiomics features, all of the features were normalized before feature selection. Each feature was subtracted by the mean value of the training group and was divided by the standard deviation value. The same normalization method was applied to the validation set. Redundant features were removed by One-way analysis of variance (ANOVA). Then, the least absolute shrinkage and selection operator (LASSO) regression method was applied to select the most distinguishable features.

Each clinical feature was assessed by univariate logistic regression. The features revealed as statistically significant with univariate logistic regression analysis were then analyzed with multivariate logistic regression analysis for model-building. A nomogram was generated for model visualization. Receiver operating characteristic (ROC) curve analyses were conducted to estimate the diagnostic performance of the models for the diagnosis of PCa.

### Statistical Analysis

All statistical analyses were performed on R software, Statistic Package for Social Science version 21, Stata 15.0, and Medcalc 15.2.2. Differences in **Table 1** were assessed by the chi-square test, the Mann-Whitney test, or *t*-test. The AUCs between different models were compared by DeLong’s test. The confidence level was set at *P* < 0.05.

## Results

A total of 203 patients were included in this study, in which 112 patients had PCa, and 91 patients had benign lesions [84 benign prostatic hyperplasia (BPH), and 7 high-grade prostatic intraepithelial neoplasis (HGPIN)]. For the lesion origin, 96 lesions originated from the peripheral zone, and 107 lesions were located in the transitional zone. The PCa had a larger size than non-PCa (long axis, 2.22 ± 1.08 mm *vs.* 1.56 ± 0.88 mm, *P* = 0.001). As shown in **Table 1**. As for the reference standard, 82 patients with benign lesions did not undergo radical prostatectomy, and the pathological results were determined by TRUS-guided biopsy.

### Feature Selection and Model-Building

**Figure 2:**
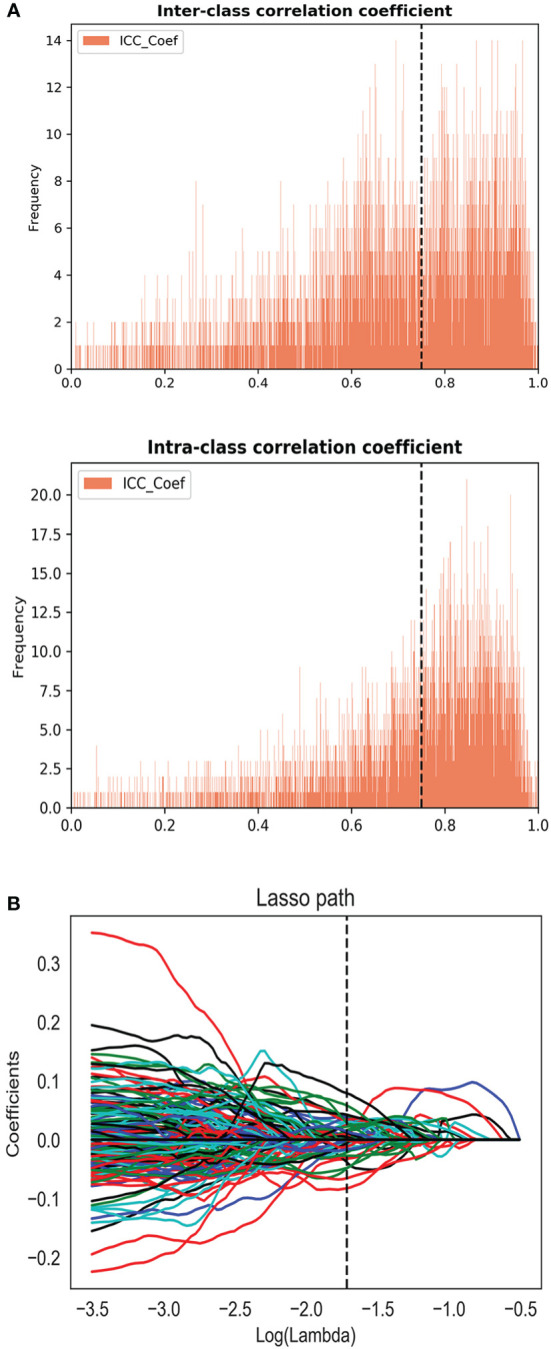
**(A)** The inter and intra-class correlation analysis. **(B)** Feature selection using the least absolute shrinkage and selection operator regression method.

For the consistency test of VOIs, the number of features with ICC > 0.75 were 522 for DWI, 655 for ADC, 471 for DCE, and 266 for T2WI, as shown in **Figure 2A**. A total of 45 features (5, 19, 10, and 11 features were extracted from T2WI, DWI, ADC, and DCE images, respectively) were selected by LASSO method (**Figure 2B**). These features all had high ICC (> 0.75). The radiomics model (Rad-score) was comprised of these features in a formula shown in **Supplementary Material**. Rad-score had statistical difference between PCa and non-PCa groups (Rad-score = 0.85 ± 0.29 *vs.* 0.12 ± 0.25, *P* < 0.001). Then the combined models were built by combining PI-RADS (odds ratio [OR] = 6.4, *P* = 0.001) with Rad-score (OR = 14.5, *P* < 0.001) or PSA using multivariate logistic regression analysis.

**Figure 3:**
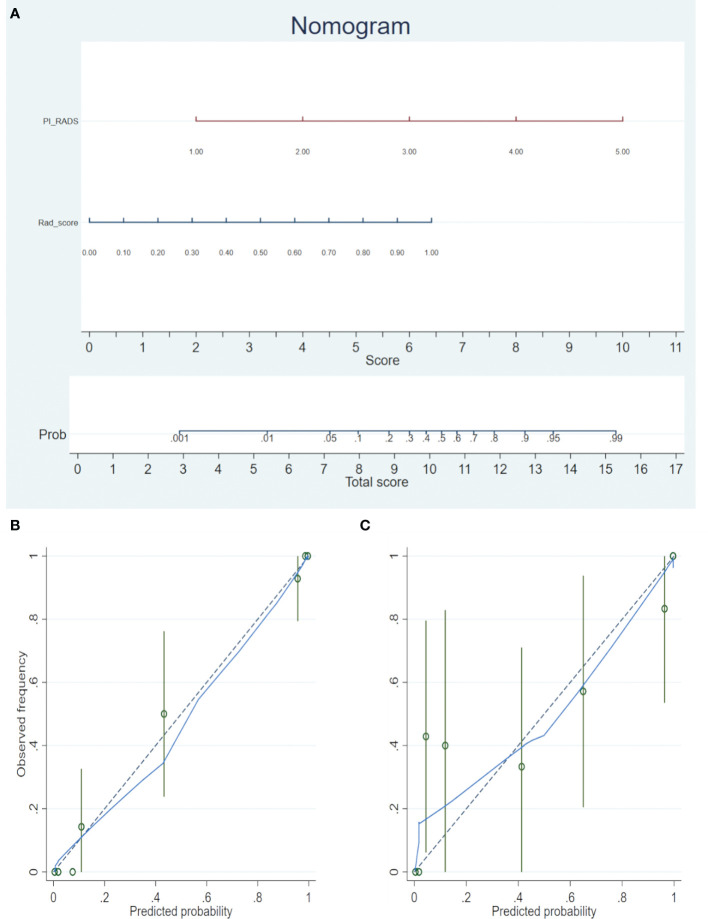
**(A)** The radiomics nomogram was developed in the training cohort, with the Rad-score and PI-RADS incorporated. **(B)** Calibration curve of the nomogram in the training set. **(C)** Calibration curve of the nomogram in the validation set. Calibration curves depict the calibration of the model in terms of the agreement between the predicted risks of PCa (the x-axis) and observed outcomes of PCa (the y-axis). The blue solid line represents the performance of the nomogram (Note: a closer fit to the diagonal dotted line represents a better prediction).

A nomogram was generated for the combined model (PI-RADS + Rad-score) visualization (**Figure 3A**). To use the nomogram, find the point for each feature on the corresponding axis, add the points for all features, and draw a line from the total points axis to the risk axis to determine the risk of PCa. Higher total score was associated with greater risk of PCa. The model yielded satisfactory fit measurement based on the training set (Hosmer-Lemeshow test, *P* = 0.943). Moreover, there were also good calibration curves for the risk estimation (**Figures 3B, C**).

### PCa *vs.* Non-PCa Classification

**Figure 4:**
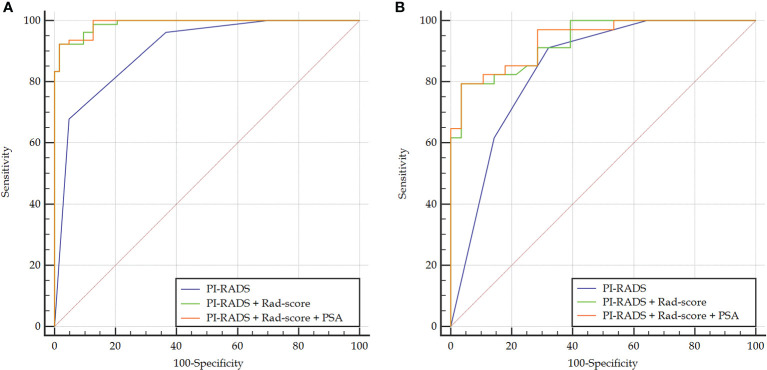
Comparison of ROC curves between PI-RADS v2.1 and the combined models in the training **(A)** and validation **(B)** sets. The combined models both had significantly higher AUCs than PI-RADS.

Rad-score had a significantly higher AUC [0.979 (95% CI, 0.940–0.996)] than PI-RADS [0.905 (0.844–0.948), *P* = 0.002] in the training set. However, the AUC between them was insignificant in the validation set [0.861 (0.749–0.936) vs. 0.845 (0.731–0.924), *P* = 0.825]. When Rad-score was added to PI-RADS, the performance of PI-RADS was significantly improved for the PCa diagnosis (AUC = 0.989, *P* < 0.001 for the training set and AUC = 0.931, *P* = 0.038 for the validation set). As shown in **Table 2** and **Figure 4**.

**Table 2:** ROC analyses of PI-RADS v2.1, Rad-score, and the combined models in the training and validation cohorts.

Model	The training cohort			*P* value	The validation cohort			*P* value
	AUC	SEN	SPE		AUC	SEN	SPE	
PI-RADS*	0.905	96.2%	63.5%		0.845	91.2%	67.9%	
Rad-score	0.979	89.7%	95.2%	0.002	0.861	82.4%	82.1%	0.825
PI-RADS + Rad-score	0.989	92.3%	98.4%	<0.001	0.931	79.4%	96.4%	0.038
PI-RADS + Rad-score + PSA	0.990	92.3%	98.4%	<0.001	0.937	79.4%	96.4%	0.026

*When PI-RADS v2.1 score of the lesion ≥ 4, this lesion was diagnosed as PCa.

P value, compared with PI-RADS.

AUC, Area under the curve; SEN, sensitivity; SPE, specificity.

In the case of clinical features, PSA, location, size, and extracapsular invasion were selected by univariate logistic regression analysis. However, only PSA was included in the combined model by multivariate logistic regression analysis. However, the addition of PSA to the combined model failed to show incremental diagnostic value (AUC = 0.990 *vs.* 0.989 for the training set; AUC = 0.937 *vs.* 0.931 for the validation set). As shown in **Table 2** and **Figure 4**.

For differentiating the peripheral zone lesions, the AUC of the combined model was higher than that of PI-RADS (AUC = 0.995 *vs.* 0.891, *P* = 0.002 for the training cohort; AUC = 0.941 vs. 0.753, *P* = 0.046 for the validation cohort; AUC = 0.987 *vs.* 0.860, *P* < 0.001 in the whole cohort). For differentiating lesions in the transitional zone, the AUC of the combined model was higher than that of PI-RADS (AUC = 0.981 *vs.* 0.909, *P* = 0.007 for the training cohort; AUC = 0.926 *vs.* 0.861, *P* = 0.170 in the validation cohort; AUC = 0.960 *vs.* 0.894, *P* = 0.006 in the whole cohort).

### Classification of PI-RADS 3 Lesions

**Figure 5:**
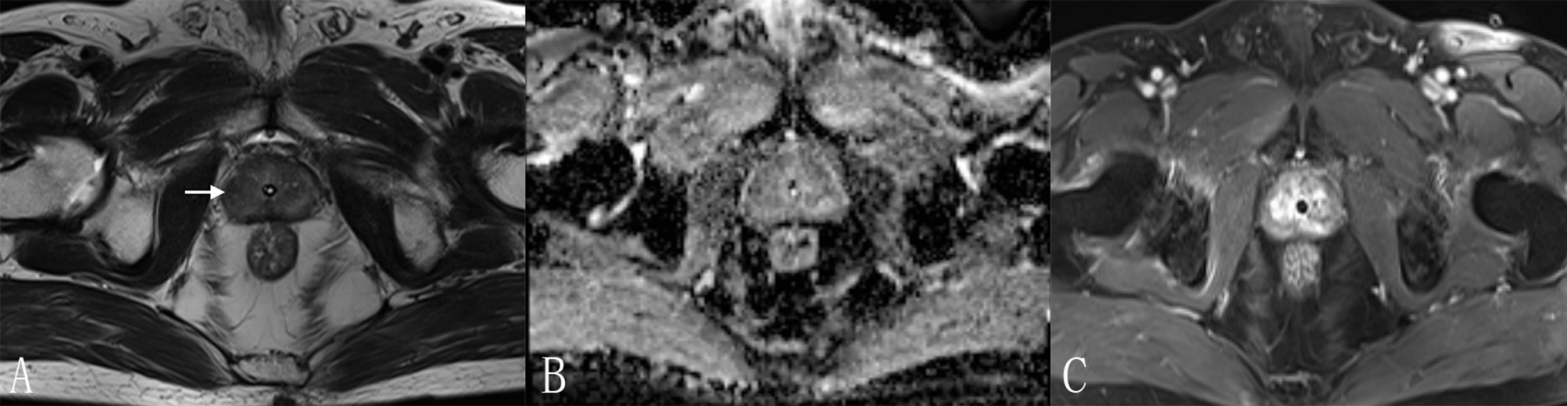
A 70-year-old man with a PI-RADS 3 lesion. **(A)** T2WI, heterogeneous signal intensity with obscured margins (arrow), which indicated 3 points according to PI-RADS v2.1; **(B)** ADC with 3 points, focal hypointense (discrete and different from the background); **(C)** DCE, contemporaneously with enhancement of adjacent normal prostatic tissues. According to **(A–C)**, this lesion was classified as PI-RADS 3, indicating this lesion had an intermediate likelihood of clinically significant cancer. However, considering Rad-score (value = 0.97) and PI-RADS (score = 3), the risk of PCa was very high (the probability was about 85%) according to the nomogram. Finally, this lesion was proven to be PCa pathologically, with Gleason score = 3 + 4.

Thirty-six patients in this study had PI-RADS 3 lesions on prostate MRI, in which 6 patients had PCa, and 30 patients had non-PCa. The Rad-score, and the combined model (Rad-score + PI-RADS) both had good diagnostic performance for the identification of PI-RADS 3 lesions (both AUC = 0.944), which was shown in **Table 3** and **Figure 5**.

**Table 3:** ROC analyses of PI-RADS, Rad-score, and the combined models for identifying PI-RADS 3 lesions.

Model	PI-RADS 3 lesions (n = 36)			*P* value
	AUC	SEN	SPE	
PI-RADS*	0.500	0	100%	
Rad-score	0.944	100%	80%	<0.001
PI-RADS + Rad-score	0.944	100%	80%	<0.001
PI-RADS + Rad-score + PSA	0.911	83.3%	90.0%	<0.001

*When PI-RADS v2.1 score of the lesion ≥ 4, this lesion was diagnosed as PCa.

P value, compared with PI-RADS.

AUC, Area under the curve; SEN, sensitivity; SPE, specificity.

## Discussion

In this study, we proposed a combined model (PI-RADS combined with Rad-score) to significantly improve the diagnostic value of PI-RADS v2.1. By adding Rad-score to PI-RADS, the combined model outperformed PI-RADS in the diagnosis of PCa (AUC = 0.989 *vs.* 0.905, *P* < 0.001 for the training set; AUC = 0.931 *vs.* 0.845, *P* = 0.038 for the validation set). Thus adding quantitative Rad-score can benefit radiologists in the diagnosis of PCa.

PI-RADS v2 is designed to improve lesion detection, localization, characterization, and risk stratification in patients with suspected cancer (15). It is known that PI-RADS v2 generally benefits from its highly structured criteria, making relatively high diagnostic sensitivity in PCa diagnosis (14). However, the specificity and inter-reader reproducibility are moderate (16–18). To address these limitations of PI-RADS v2, an updated version (PI-RADS v2.1) was developed in 2019. One of the major modifications in version 2.1 is the diagnostic criteria for the transitional zone PCa of low T2WI scores. When comparing the performance between version 2 and 2.1 for characterization of suspected PCa, several studies suggested that AUC tended to be higher in version 2.1 than in version 2 without statistical significance (6–8). It must be noted that PI-RADS v2.1 still showed a high false positive rate (moderate specificity) for PCa diagnosis, similar to that with PI-RADS v2. Moreover, PI-RADS 3 lesions are frequently encountered (22–32%), and carry a moderate malignant potential (up to 20–30%), the stratification of these lesions is still challenging when using PI-RADS (19). Therefore, quantitative parameters, such as radiomics, may help to prevent misdiagnoses and improve performance of PI-RADS v2.1.

Compared with qualitative or subjective explanation of radiological images, radiomics permits high-throughput extraction of quantitative features to evaluate the degree of intratumor heterogeneity (20). In recent years, radiomics analysis has appeared as a potent tool for constructing decision-support models. A number of studies have used radiomics analysis to automate PCa diagnosis and risk stratification (21, 22). While few studies (4, 14) focused on the comparison of the diagnostic value between radiomics and PI-RADS. Our results suggested that the AUC of radiomics was higher than that of PI-RADS in the training set, which was consistent with previous studies. However, the difference of AUC was insignificant in the validation set, which was not entirely consistent with the previous studies. This finding showed that radiomics might not replace PI-RADS currently. The building methods between our study and those of previous studies were all machine learning, and all these studies lacked external validation. However, there were still some differences that need to be explained. Firstly, the version of PI-RADS in our study was 2.1, which was different from version 2 in previous studies (4, 14). Secondly, Chen et al. (14) only used T2WI and ADC images, in which lack of enhanced images reduced the effectiveness of mpMRI and radiomics. Finally, prior studies (4, 14) had the smaller sample sizes than ours, especially Wang et al. (14).

In our study, extracapsular invasion, location of lesions, and tumor diameter did not present enough predictive power for the differentiation of benign and malignant lesions. Thus, we integrated PSA into the combined model (Rad-score + PI-RADS). However, adding PSA failed to show incremental diagnostic value. This might be because the AUC of the combined model was high enough. In our work, when Rad-score were added, the diagnostic performance of PI-RADS was prominently improved: the specificity increased from 63.5 to 98.4% in the training set, and from 67.9 to 96.4% in the validation set. Adding Rad-score to PI-RADS might overcome the challenge of moderate specificity of PI-RADS. For the individual zone-based analysis, the combined model outperformed PI-RADS in the training, validation, and whole sets for differentiating lesions in the peripheral zone. However, the combined model failed to show significantly higher diagnostic performance in differentiating transitional zone lesions for the validation cohort (*P* = 0.170). We speculated that this might be related to the small sample size of transitional zone lesions in the validation set.

For PI-RADS 3 lesions classification, our exploratory results may provide preliminary evidence to justify the use of radiomics in this field. In clinical practice of the future, the validation of radiomics is important for the challenging PI-RADS 3 lesions, including biopsy or short-term follow-up in these lesions with high risk of PCa indicated by radiomics.

Our study had several limitations. First, due to its retrospective design, there might be selection bias between PCa and non-PCa groups, and the high b-value images (b value ≥ 1,400 s/mm^2^) failed to be obtained. Second, prospective and external validation was not performed. Third, all mpMRI images were obtained from a single institution. In the future, multicenter verification is needed to extend the versatility of the experimental results.

In conclusion, although the radiomics model cannot replace PI-RADS currently, adding radiomics to PI-RADS has the potential to improve the performance of the structured PI-RADS scheme by providing radiologists with quantitative and standardized criteria, thereby enabling us to more confidentially detect prostate cancer.

## Data Availability Statement

The original contributions presented in the study are included in the article/**Supplementary Material**. Further inquiries can be directed to the corresponding author.

## Ethics Statement

The studies involving human participants were reviewed and approved by West China Hospital of Sichuan University Biomedical Research Ethics Committee. Written informed consent for participation was not required for this study in accordance with the national legislation and the institutional requirements. Written informed consent was not obtained from the individual(s) for the publication of any potentially identifiable images or data included in this article.

## Author Contributions

BS and LY conceived of the presented idea. ML and YY collected the data. ML, JX, and CH analyzed the data. ML drafted the manuscript. All authors reviewed the manuscript and BS made corrections to the manuscript. All authors contributed to the article and approved the submitted version.

## Funding

This work was supported by grants from China International Medical Foundation (2019 SKY Imaging Research Funds, NO. Z-2014-07-1912).

## Conflict of Interest

Authors JX and CH were employed by the company Beijing Deepwise & League of PHD Technology Co., Ltd.

The remaining authors declare that the research was conducted in the absence of any commercial or financial relationships that could be construed as a potential conflict of interest.

## References

[1] Cheung E Wadhera P Dorff T Pinski J. Diet and prostate cancer risk reduction. Expert Rev Anticancer Ther (2008) 8(1):43–50. 10.1586/14737140.8.1.43 18095882

[2] Boettcher AN Usman A Morgans A VanderWeele DJ Sosman J Wu JD. Past, Current, and Future of Immunotherapies for Prostate Cancer. Front Oncol (2019) 9:884. 10.3389/fonc.2019.00884 31572678 PMC6749031

[3] Tian JY Guo FJ Zheng GY Ahmad A. Prostate cancer: updates on current strategies for screening, diagnosis and clinical implications of treatment modalities. Carcinogenesis (2018) 39(3):307–17. 10.1093/carcin/bgx141 29216344

[4] Chen T Li M Gu Y Zhang Y Yang S Wei C. Prostate Cancer Differentiation and Aggressiveness: Assessment With a Radiomic-Based Model vs. PI-RADS v2. J Magn Reson Imag (2019) 49(3):875–84. 10.1002/jmri.26243 PMC6620601 30230108

[5] Hoffmann R Logan C O’Callaghan M Gormly K Chan K Foreman D. Does the Prostate Imaging-Reporting and Data System (PI-RADS) version 2 improve accuracy in reporting anterior lesions on multiparametric magnetic resonance imaging (mpMRI)? Int Urol Nephrol (2018) 50(1):13–9. 10.1007/s11255-017-1753-1 29188489

[6] Tamada T Kido A Takeuchi M Yamamoto A Miyaji Y Kanomata N. Comparison of PI-RADS version 2 and PI-RADS version 2.1 for the detection of transition zone prostate cancer. Eur J Radiol (2019) 121:108704. 10.1016/j.ejrad.2019.108704 31669798

[7] Xu L Zhang G Zhang D Zhang X Bai X Yan W. Comparison of PI-RADS version 2.1 and PI-RADS version 2 regarding interreader variability and diagnostic accuracy for transition zone prostate cancer. Abdom Radiol (NY) (2020) 45(12):4133–41. 10.1007/s00261-020-02738-6 32918577

[8] Byun J Park KJ Kim MH Kim JK. Direct Comparison of PI-RADS Version 2 and 2.1 in Transition Zone Lesions for Detection of Prostate Cancer: Preliminary Experience. J Magn Reson Imag (2020) 52(2):577–86. 10.1002/jmri.27080 32045072

[9] Bjurlin MA Carroll PR Eggener S Fulgham PF Margolis DJ Pinto PA. Update of the Standard Operating Procedure on the Use of Multiparametric Magnetic Resonance Imaging for the Diagnosis, Staging and Management of Prostate Cancer. J Urol (2020) 203(4):706–12. 10.1097/JU.0000000000000617 PMC8274953 31642740

[10] Zhang L Tang M Chen S Lei X Zhang X Huan Y. A meta-analysis of use of Prostate Imaging Reporting and Data System Version 2 (PI-RADS V2) with multiparametric MR imaging for the detection of prostate cancer. Eur Radiol (2017) 27(12):5204–14. 10.1007/s00330-017-4843-7 28656462

[11] Mussi TC Yamauchi FI Tridente CF Tachibana A Tonso VM Recchimuzzi DR. Interobserver Agreement and Positivity of PI-RADS Version 2 Among Radiologists with Different Levels of Experience. Acad Radiol (2019) 26(8):1017–22. 10.1016/j.acra.2018.08.013 30268722

[12] Chen F Cen S Palmer S. Application of Prostate Imaging Reporting and Data System Version 2 (PI-RADS v2): Interobserver Agreement and Positive Predictive Value for Localization of Intermediate- and High-Grade Prostate Cancers on Multiparametric Magnetic Resonance Imaging. Acad Radiol (2017) 24(9):1101–6. 10.1016/j.acra.2017.03.019 28546032

[13] Avanzo M Stancanello J El Naqa I. Beyond imaging: The promise of radiomics. Phys Med (2017) 38:122–39. 10.1016/j.ejmp.2017.05.071 28595812

[14] Wang J Wu CJ Bao ML Zhang J Wang XN Zhang YD. Machine learning-based analysis of MR radiomics can help to improve the diagnostic performance of PI-RADS v2 in clinically relevant prostate cancer. Eur Radiol (2017) 27(10):4082–90. 10.1007/s00330-017-4800-5 28374077

[15] Hassanzadeh E Glazer DI Dunne RM Fennessy FM Harisinghani MG Tempany CM. Prostate imaging reporting and data system version 2 (PI-RADS v2): a pictorial review. Abdom Radiol (NY) (2017) 42(1):278–89. 10.1007/s00261-016-0871-z PMC5247306 27522352

[16] Smith CP Türkbey B. PI-RADS v2: Current standing and future outlook. Turk J Urol (2018) 44(3):189–94. 10.5152/tud.2018.12144 PMC5937636 29733790

[17] Kasel-Seibert M Lehmann T Aschenbach R Guettler FV Abubrig M Grimm MO. Assessment of PI-RADS v2 for the Detection of Prostate Cancer. Eur J Radiol (2016) 85(4):726–31. 10.1016/j.ejrad.2016.01.011 26971415

[18] Rosenkrantz AB Ginocchio LA Cornfeld D Froemming AT Gupta RT Turkbey B. Interobserver Reproducibility of the PI-RADS Version 2 Lexicon: A Multicenter Study of Six Experienced Prostate Radiologists. Radiology (2016) 280(3):793–804. 10.1148/radiol.2016152542 27035179 PMC5006735

[19] Giambelluca D Cannella R Vernuccio F Comelli A Pavone A Salvaggio L. PI-RADS 3 Lesions: Role of Prostate MRI Texture Analysis in the Identification of Prostate Cancer. Curr Probl Diagn Radiol (2019) 50(2):175–85. 10.1067/j.cpradiol.2019.10.009 31761413

[20] Li M Zhu YZ Zhang YC Yue YF Yu HP Song B. Radiomics of rectal cancer for predicting distant metastasis and overall survival. World J Gastroenterol (2020) 26(33):5008–21. 10.3748/wjg.v26.i33.5008 PMC7476170 32952346

[21] Stanzione A Gambardella M Cuocolo R Ponsiglione A Romeo V Imbriaco M. Prostate MRI radiomics: A systematic review and radiomic quality score assessment. Eur J Radiol (2020) 129:109095. 10.1016/j.ejrad.2020.109095 32531722

[22] Patel N Henry A Scarsbrook A. The value of MR textural analysis in prostate cancer. Clin Radiol (2019) 74(11):876–85. 10.1016/j.crad.2018.11.007 30573283

