# Supplementary material for: Use of Radiomics to Improve Diagnostic Performance of PI-RADS v2.1 in Prostate Cancer
Source: Front Oncol. 2021 Feb 17;10:631831. doi: 10.3389/fonc.2020.631831 (PMC7925826; doi:10.3389/fonc.2020.631831)
Supplement: Supplementary file 1 [file DataSheet_1.docx]

Supplementary Material

# Supplementary Data

Rad-score = 1/{1+EXP(-(gradient_glrlm_RunEntropy@dce × 0.0426 + original_glszm_LargeAreaHighGrayLevelEmphasis@dwi × 0.5376 + square_gldm_GrayLevelVariance@dwi × 0.0522 + square_gldm_SmallDependenceHighGrayLevelEmphasis@dwi × 0.1241 + square_glrlm_ShortRunLowGrayLevelEmphasis@dwi × -0.3369 + exponential_glrlm_RunLengthNonUniformityNormalized@adc × -0.0659 + wavelet-LLH_glcm_Idn@dce × 0.491 + wavelet-LHL_glcm_InverseVariance@t2wi × 0.8018 + gradient_glcm_Idn@dce × 0.0454 + wavelet-LHL_glcm_Correlation@dwi × 0.1349 + wavelet-LHL_glcm_Imc1@dce × 0.314 + wavelet-LLL_glcm_Contrast@dwi × 0.2949 + gradient_firstorder_Variance@dce × 0.1869 + logarithm_glcm_InverseVariance@t2wi × 0.3192 + exponential_glcm_DifferenceVariance@dwi × 0.136 + exponential_gldm_LargeDependenceLowGrayLevelEmphasis@dwi × -0.0478 + wavelet-LHH_glcm_InverseVariance@t2wi × 0.3235 + square_gldm_DependenceNonUniformityNormalized@dwi × 0.2845 + wavelet-HLL_gldm_DependenceNonUniformityNormalized@dce × -0.7058 + logarithm_glcm_DifferenceAverage@dwi × -0.3209 + square_gldm_DependenceVariance@dwi × -0.5986 + exponential_glrlm_ShortRunEmphasis@adc × -0.9012 + square_glcm_Imc2@adc × -0.6034 + square_gldm_LargeDependenceHighGrayLevelEmphasis@t2wi × 0.1442 + square_glrlm_LongRunEmphasis@adc × 0.4506 + wavelet-LHH_firstorder_InterquartileRange@dce × 0.5869 + wavelet-HLL_glszm_SizeZoneNonUniformityNormalized@dce × -0.4755 + wavelet-HHL_firstorder_Range@adc × 0.773 + square_gldm_GrayLevelNonUniformity@dce × 0.1441 + wavelet-HHL_glcm_Correlation@adc × -0.5905 + wavelet-HLH_gldm_LargeDependenceLowGrayLevelEmphasis@dwi × -0.1354 + lbp-2D_firstorder_Skewness@dwi × 0.1644 + lbp-2D_firstorder_RobustMeanAbsoluteDeviation@dwi × -0.3213 + wavelet-LLH_firstorder_10Percentile@dce × -0.5949 + gradient_glcm_Idn@adc × -0.382 + wavelet-LLH_glszm_ZoneVariance@dwi × -0.5065 + wavelet-HLL_gldm_SmallDependenceHighGrayLevelEmphasis@dwi × -0.0955 + gradient_glszm_LargeAreaLowGrayLevelEmphasis@adc × 0.3388 + wavelet-LLH_glszm_LargeAreaEmphasis@dwi × -0.3084 + wavelet-LLL_gldm_DependenceNonUniformityNormalized@dwi × 0.5048 + wavelet-HLH_glszm_GrayLevelVariance@adc × 0.4015 + wavelet-LHL_firstorder_InterquartileRange@dce × 0.4771 + wavelet-LHH_glcm_DifferenceEntropy@t2wi × -0.6079 + wavelet-HHH_firstorder_RobustMeanAbsoluteDeviation@adc × 0.7475 + wavelet-HHL_glszm_LargeAreaEmphasis@dwi × -0.239+0.6570813))}

# Supplementary Tables

| The parameters of multi-parametric MRI sequences | | | | |
| --- | --- | --- | --- | --- |
| sequence type | repetition time / echo time (TR/TE) | section thickness | field of view (FOV) | Bandwidth (Hz/Px) |
| Axial T2WI | 6220ms/104ms | 3.0 mm | 200 mm | 200 |
| DWI | 4200 ms/74ms | 3.0 mm | 200 mm | 1202 |
| DCE | 3.9ms/1.9ms | 2.0 mm | 376 mm | 450 |
